# Macrophage Function in Calcium Oxalate Kidney Stone Formation: A Systematic Review of Literature

**DOI:** 10.3389/fimmu.2021.673690

**Published:** 2021-05-24

**Authors:** Kazumi Taguchi, Atsushi Okada, Rei Unno, Shuzo Hamamoto, Takahiro Yasui

**Affiliations:** Department of Nephro-urology, Nagoya City University Graduate School of Medical Sciences, Nagoya, Japan

**Keywords:** urolithiasis, nephrocalcinosis, calcium oxalate (CaOx), Randall plaque, macrophage, M1-macrophage, M2-macrophage, monocyte

## Abstract

**Background:**

The global prevalence and recurrence rate of kidney stones is very high. Recent studies of Randall plaques and urinary components *in vivo*, and *in vitro* including gene manipulation, have attempted to reveal the pathogenesis of kidney stones. However, the evidence remains insufficient to facilitate the development of novel curative therapies. The involvement of renal and peripheral macrophages in inflammatory processes offers promise that might lead to the development of therapeutic targets. The present systematic literature review aimed to determine current consensus about the functions of macrophages in renal crystal development and suppression, and to synthesize evidence to provide a basis for future immunotherapy.

**Methods:**

We systematically reviewed the literature during February 2021 according to the Preferred Reporting Items for Systematic Reviews and Meta-Analyses (PRISMA) guidelines. Articles investigating the relationship between macrophages and urolithiasis, particularly calcium oxalate (CaOx) stones, were extracted from PubMed, MEDLINE, Embase, and Scopus. Study subjects, languages, and publication dates were unrestricted. Two authors searched and screened the publications.

**Results:**

Although several studies have applied mixed modalities, we selected 10, 12, and seven (total, n = 29) of 380 articles that respectively described cultured cells, animal models, and human samples.

The investigative trend has shifted to macrophage phenotypes and signaling pathways, including micro (m)-RNAs since the discovery of macrophage involvement in kidney stones in 1999. Earlier studies of mice-associated macrophages with the acceleration and suppression of renal crystal formation. Later studies found that pro-inflammatory M1- and anti-inflammatory M2-macrophages are involved. Studies of human-derived and other macrophages *in vitro* and *ex vivo* showed that M2-macrophages (stimulated by CSF-1, IL-4, and IL-13) can phagocytose CaOx crystals, which suppresses stone development. The signaling mechanisms that promote M2-like macrophage polarization toward CaOx nephrocalcinosis, include the ﻿NLRP3, PPARγ-miR-23-Irf1/Pknox1, miR-93-TLR4/IRF1, and miR-185-5p/CSF1 pathways.

Proteomic findings have indicated that patients who form kidney stones mainly express M1-like macrophage-related proteins, which might be due to CaOx stimulation of the macrophage exosomal pathway.

**Conclusions:**

This systematic review provides an update regarding the current status of macrophage involvement in CaOx nephrolithiasis. Targeting M2-like macrophage function might offer a therapeutic strategy with which to prevent stones *via* crystal phagocytosis.

## Introduction

The prevalence of kidney stones has increased worldwide, and its high recurrence rate is also a factor that affects medical and economic resources (1–4). Considerable research effort has been directed toward finding a cure, but the pathology of kidney stone formation is complex and awaits elucidation despite recent technological innovations. Kidney stones are recognized as a multifactorial disease similar to metabolic syndrome (MetS) (5, 6). Renal function, mineral and lipid metabolism, inflammatory processes, oxidative stress, and insulin resistance can cause calcium oxalate (CaOx) crystals to develop (7).

Most kidney stones consist of calcium oxalate (CaOx) (8, 9). The hypotheses presented to account for the pathogenesis of CaOx stones are free- and fixed-particle mechanisms (10); the latter is also known as Randall plaques (RP) that comprise apatite formed by calcium phosphate that grow in the interstitial space around the loop of Henle (11). In contrast to stones that develop within the tubular lumen or renal collecting system, crystal precursors of RP are surrounded by other molecular and cellular structures, which might be influenced by impaired homeostasis (12, 13). Among such lithogenic environments, chemical and mineral component overload or other sources of inflammatory stimulation might act as first triggers that are followed by reactive oxygen species (ROS), which subsequently induce renal epithelial cell damage resulting in CaOx crystal deposition (14). The primary defense mechanism against such cellular impairment is autophagy involving endocytosis (15), and a secondary defense mechanism extends to peri-tubular cells in the interstitial space and immune cells from the circulation (16). The innate defense system that clears crystal deposits from renal tissues is key to finding a fundamental solution for developing novel treatments for kidney stones.

Understanding the role of macrophages (Mφ) in renal crystal formation can help to identify a solution. Renal or peripheral Mφ involvement in kidney stone disease was first reported by de Water et al. in 1999 (17). They discovered that Mφs migrate to crystal deposition sites and engulf the crystals. Given M1 pro- and M2 anti-inflammatory (18) polarization, the involvement of Mφ in renal crystal formation is probably diversified in different ways. Because the ability to phagocytose crystals is greater for M2- than M1-like Mφs (19), regulating their polarization might have therapeutic value (20). Much about the clinical application of Mφ to preventing kidney stone development has been reported over the last two decades (21).

This systematic review aimed to provide collective evidence of Mφ function in renal crystal development and suppression, and to provide ideas for the future direction of Mφ research into the clinical application of Mφ immunotherapy.

## Materials and Methods

This systematic literature review was conducted in accordance with the Preferred Reporting Items for Systematic Reviews and Meta-Analyses (PRISMA) guidelines (22).

### Search Strategy

We searched PubMed, MEDLINE, Embase, and Scopus databases using the MeSH key terms, “macrophages” OR “macrophage”, OR “monocyte”, AND “urolithiasis”, OR “calcium oxalate”, OR “kidney stone”. Article type, publication date, language, or species were not restricted during the initial search.

### Eligibility Criteria

Articles that focused on the relationship between Mφ and monocyte function and the pathogenesis of urolithiasis were included. Articles describing findings of inflammatory cytokines/chemokines secreted by Mφs and monocytes were excluded because direct connections with Mφs and/or monocytes were limited. Since we focused on experimental and translational findings, original articles were favored over reviews or commentary articles, which referred to and/or summarized findings published by others.

### Data Collection and Description

Two authors (KT and RU) independently reviewed the titles and abstracts of the articles identified in the initial search during February 8^th^, 2021. Data were extracted from articles that met the eligibility criteria and reassessed in full-text articles during primary and secondary screening, respectively. The most recent of duplicate published articles was included. Disagreements and discrepancies between the two screens were resolved through discussion and consensus with the other authors. The following data were extracted from all eligible full-text articles: first author, journal name, year of publication, type of study, methodology, type of experimental sources, and main findings concerning Mφ/monocyte function.

The Mφ phenotypes were reported in accordance with the latest nomenclature, experimental guidelines (18), and reviews regarding organ-specific findings (20, 23, 24).

## Results

**Figure 1:**
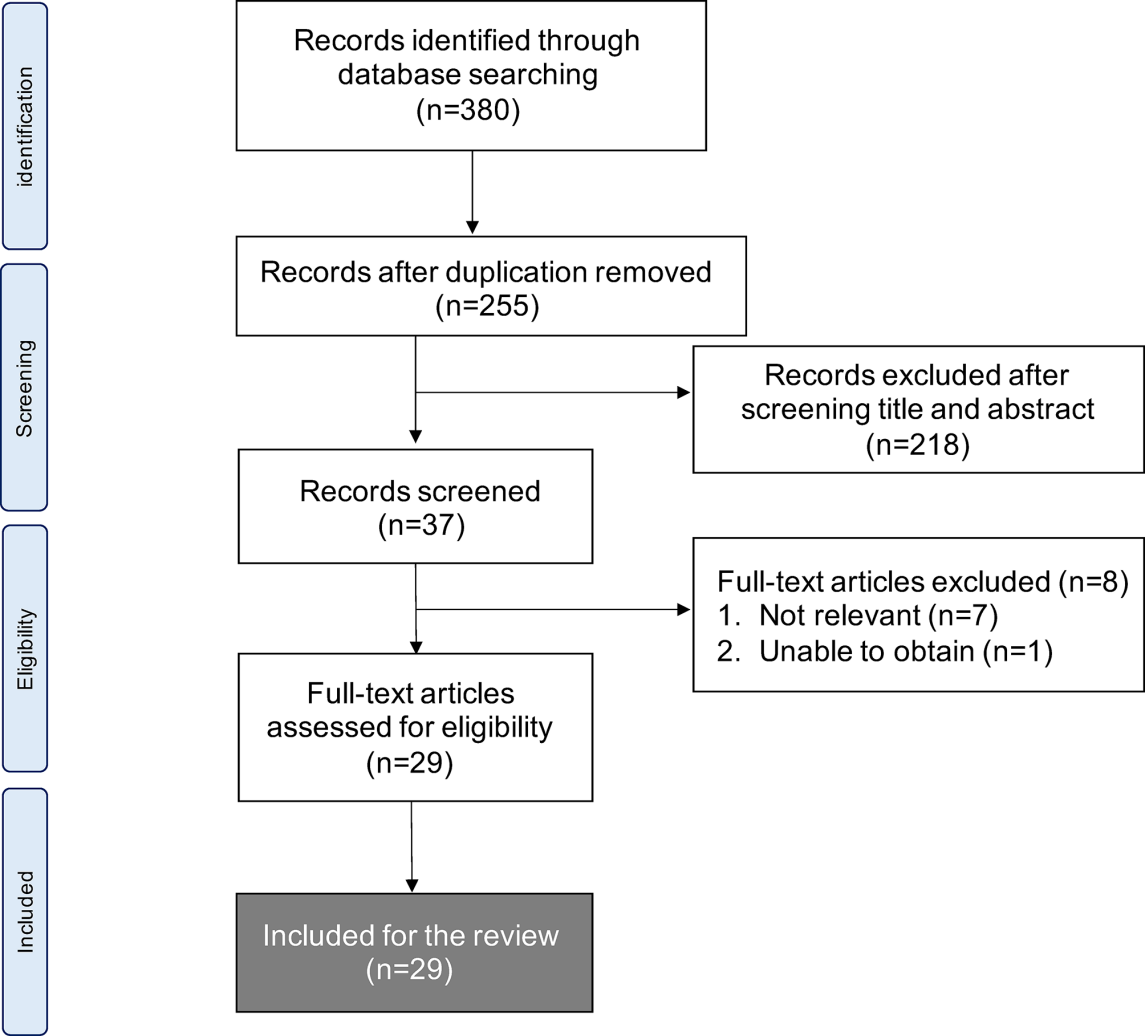
Flow chart of methods used to conduct systematic literature review in accordance with PRISMA guidelines.

We identified 380 articles that met the search strategy and criteria across all databases in the initial literature search. After the first screen of titles and abstracts, the second screen of full texts filtered them into 29 articles that were eligible for review. **Figure 1** shows the PRISMA flowchart.

**Table 1** summarizes the findings of 29 articles describing various studies that were published in China, Germany, Japan, the Netherlands, Thailand, and the USA between 1999 and 2020. These articles described mostly translational research studies including rat and mouse models of nephrocalcinosis caused by hyperoxaluria *in vivo*, murine, canine, and human renal tubular epithelial cell lines *in vitro*, and human renal papillar, peripheral blood, and urine samples *ex vivo*.

**Table 1:** Summary of systematically reviewed literature.

First Author	Year	Journal	*In vitro*	*In vivo*	Human tissues	Mφ phenotypes *in vitro, ex vivo*, and in vivo*	Main findings
**de Water R**	1999	Am J Kidney Dis		Wistar rats with EG/AC	Renal tissue from patients with oxalosis	M(-)/NA/NA M1: NA M2: NA	ED1 and CD68 positive Mφ around the crystals
**de Water R**	2000	Am J Kidney Dis		Wistar rats with EG/AC		M(-)/NA/NA M1: NA M2: NA	ED1-positive Mφ expression (time course)/crystal encapsulation by multinucleated cells
**de Water R**	2001	Am J Kidney Dis	I9.1			M(-)/NA/NA M1: NA M2: NA	Time course dissolution of internalized crystals by Mφ
**Okada A**	2009	J Bone Miner Res		C57BL/6N mice with GOX(IAI)		M(-)/NA/NA M1: NA M2: NA	Transcriptome Mφ activation is related to spontaneous renal crystal elimination
**Okada A**	2010	J Bone Miner Res		C57BL/6N mice with GOX(IAI)		M(-)/NA/NA M1: NA M2: NA	Pathway validation of relationship between renal crystal deposition and Mφ-related genes/Mφ phagocytosis of crystals
**Singhto N**	2010	J Proteome Res	U937			M(-)/NA/NA M1: NA M2: NA	COM crystals cause increased cellular apoptosis and survival including protein synthesis/stability, mRNA stability, and lipid metabolism
**Singhto N**	2013	J Proteome Res	Mφ from U937			M(-)/NA/NA M1: NA M2: NA	Interaction between HSP90 and F-actin for phagocytosis and migration of Mφ toward COM crystals
**Zuo L**	2014	J Urol	RAW264.7, M-1, 3T3/L1			M(-)/NA/NA M1: NA M2: NA	Co-culture of M-1, RAW264.7, and 3T3-L1 increase gene expression of OPN, MCP-1, and TNFα, that increases COM crystal adhesion on M-1 cells
**Taguchi K**	2014	J Am Soc Nephrol	Murine BMDM, M-1	CSF-1 deficient mice with GOX(IAI)		M(-)/M(LPS)/M(IL-4) M1: CD11c, Ly6C M2: CD163, CD206	CSF-1-deficient mice have fewer M2-like Mφs, resulting in increased renal crystal deposition/M(IL-4) have more phagocytosis capacity than M(LPS)
**Taguchi K**	2015	J Urol		Leptin deficient mice with EG+HFD		NA/NA/NA M1: Ccl5, Ccr7, CD11c, Il23a, Il6, Il10, Ly6C, Nos2, Tnf M2: Arg1, Ccl24, Ccr2, CD163, CD206, Chi3l3, Pparg	Renal M1-like Mφs with crystal deposition are increased in leptin-deficient MetS model sunder hyperoxaluria and hyperlipidemia.
**Taguchi K**	2016	Sci Rep	Murine BMDM, M-1	C57BL/6J mice with GOX((IAI)	Renal papillae from CaOx-/non-stone formers	M(-)/M(LPS+IFNγ)/M(IL-4+IL-13) M1: CCR2, CD11c, Il6, Il10, IL-10, Ly6C, Nos2, NOS2, Tnf, TNF M2: Arg1, CD163, CD206, Chi3l3, Il4, PPARG, Retnla	M(IL-4+IL-13) attenuates while M(LPS+IFNγ) facilitates renal CaOx crystal formation/low M2-like Mφ, whereas M1-like Mϕ-related genes are expressed in papillary tissues of CaOx stone formers.
**Williams J**	2016	Urology			Blood samples from CaOx stone formers/controls	M(-)/NA/NA M1: NA M2: NA	Monocyte mitochondrial function is decreased in CaOx stone formers
**Kusmartsev S**	2016	J Urol			Human Mφ-derived from buffy coat samples	M(-)/M(GM-CSF)/M(CSF-1) M1: NA M2: NA	Ability to phagocytose CaOx stones is greater in M(M-CSF) than M(GM-CSF) and this is mediated through clathrin
**Chiangjong W**	2016	Sci Rep	U937, MDCK			M(-)/NA/NA M1: NA M2: NA	Activation of monocytic cell migration by COM crystal-binding protein, enolase-1
**Taguchi K**	2017	J Am Soc Nephrol			Renal papillae from CaOx-/non-stone formers	NA/NA/NA M1: NA M2: NA	Activity and numbers of immune cells including Mφ and plasma cells are increased in RP papillary mucosa
**Anders HJ**	2018	Kidney Int		Nlrp3- and Asc-deficient mice with a sodium oxalate diet		NA/NA/NA M1: IL1b M2: CD206, TGFβ	NLRP3 inhibition shifts M1-like Mφ to M2-like Mφ and suppressed of CaOx nephrocalcinosis-related renal fibrosis
**Singhto N**	2018	Front Immunol	U937, Mφ from U937, Jurkat			M(-)/NA/NA M1: NA M2: NA	COM-treated macrophage exosomes enhance monocyte activity and migration, and macrophage phagocytic activity
**Singhto N**	2018	J Proteomics	Mφ from U937, MDCK			M(-)/NA/NA M1: NA M2: NA	COM-treated Mφ exosomes are fragile and trigger MDCK cells to secrete more IL-8.
**Dominguez-Gutierrez PR**	2018	Front Immunol	THP-1		Mφ and monocytes derived from buffy coat samples	M(-)/M(GM-CSF)/M(CSF-1) M1: CD68, CD86, Ifng, Il12, Tnf M2: CD163, CD206, Ifna2a, Ifnb, Il10	CaOx crystal-treated Mφ express M1-like phenotype; supernatants from CaOx-treated monocytes enhance M(CSF-1) crystal phagocytosis.
**Yu J**	2018	Urolithiasis	M1Mφ from U937, HK-2			M(-)/M(LPS)/M(IL-4) M1: CCL2, TNFα M2: TGFβ	Increased oxidative stress, MCP-1, and OPN expression in COM-treated HK-2 by M(LPS).
**Liu Q**	2019	Kidney Blood Press Res	THP-1, HK-2 cells			M(-)/M(LPS+ IFNγ)/M(IL-4+IL-13) M1: IL-1β, IL-6, TNFα M2: IL-1ra, CD206, IL-10, TGFβ	Protective role of M(IL-4+IL-13) against oxidative stress damage and apoptosis is *via* inhibition of NAPDH oxidase-ROS-p38 MAPK pathway.
**Kusumi K**	2019	Urolithiasis			Urine from kidney stone forming children/controls	NA/NA/NA M1: NA M2: NA	Levels of MIP1β and IL-13 are significantly higher in patients with kidney stones than in controls.
**Okada A**	2019	Clin Exp Nephrol			Urine from CaOx stone formers/non-stone formers	NA/NA/NA M1: NA M2: NA	IL-1a, IL-1b, IL-4, IL-10, and GM-CSF have potential as biomarkers for differentiating individuals with and without urinary stones
**Okada A**	2019	Kidney Blood Press Res	J774.1			M(-)/NA/NA M1: NA M2: NA	Diachronic elimination of engulfed COM crystals in in Mφ lysosomes
**Xi J**	2019	J Cell Physiol	HK-2	C57BL/6J mice with GOX(IAI)	Peripheral blood from CaOx stone formers	M(-)/M(LPS+ IFNγ)/M(IL-4) M1: Ccr2, Il1b, Nos2 M2: Arg1, CD163, Il10	SIRT3 suppresses CaOx crystal formation by promoting M2-like Mφ *via* FOXO1 deacetylation
**Zhu W**	2019	Cell Death Dis	THP-1, RAW264.7, HK-2, M-1, HEK293	Cdh16-ARKO mice with GOX(IAI)/SD rats with HLP		M(-)/NA/NA M1: Nos1, Ccr7, Il6, Irf5, Tnf M2: Arg1, Ccl22, CD163, CD206, Il10	AR alters Mφ recruitment and M2-like polarization to influence CaOx crystal deposition *via* miRNA-185-5p/CSF-1 signaling
**Chen Z**	2019	Am J Physiol Renal Physiol	Murine BMDM	C57BL/6 mice with GOX(IAI)		M(-)/M(LPS)/M(IL-4) M1: CD11c, Il1b, Il6, Nfkb, Nos2, pSTAT1, Tnf M2: Arg1, CD163, CD206, Il4, Il10	Pioglitazone protects against CaOx crystal formation by promoting M2-like Mφ polarization through PPARγ-miR-23-Irf1/Pknox1 axis
**Liu H**	2020	Theranostics	Murine BMDM, RTEC	C57BL/6J mice with GOX(IAI)		M(-)/NA/NA M1: CD11c, Il1b, Il6, Nos2, Tnf M2: Arg1, CD206, Il10	Enhanced M1-like Mφ polarization and renal injury *via* Nrf2-miR-93-TLR4/IRF1 axis leads to CaOx crystal formation
**Yang X**	2020	Theranostics	Murine BMDM, RTEC	C57BL/6J mice with GOX(IAI)		M(-)/NA/NA M1: Il1b, Il6, Nos2, Tnf M2: Arg1, Chi3l3, Il10, Retnla	Attenuation of CaOx crystal development and renal injury by M2 to M1 shift *via* AhR-miR-142a-IRF1/HIF-1α axis

*The first row describes the in vitro and ex vivo characteristics of macrophages (non-polarized/M1-like/M2-like) in accordance with the nomenclature published in 2014 (Murray et al. Immunity). The 2^nd^ and 3^rd^ rows describe the markers used for M1- and M2-like detection in the literature, respectively.

Cell lines and animals: 3T3/L1, murine adipocytes; Cdh16-ARKO, renal tubule-specific androgen receptor knockout; HEK-293, human embryonic kidney cells; HK-2, human proximal tubule epithelial cells; I9.1, murine macrophages; J774.1, murine macrophages; Jurkat, human T lymphocytes; M-1, murine collecting duct; MDCK, canine renal tubular epithelial cells; RAW264.7, murine macrophages; SD rats, Sprague Dawley rats; THP-1, human monocytes; U937, human monocytes.

AC, ammonium chloride; AR, androgen receptor; Arg1, Arginase 1; ASC, apoptosis-associated speck-like protein; BMDM, bone marrow derived macrophages; Ccl, C-C motif chemokine ligand; Ccr, C-C chemokine receptor; Chi3l3, Chitinase 3-like 3; COM, calcium oxalate monohydrate; CSF-1, colony stimulating factor-1; EG, ethylene glycol; FOXO1, forkhead box O1; GM-CSF, granulate macrophage colony stimulating factor-1; HFD, high fat diet; HLP, hydroxy-L-proline; HSP, heat shock protein; IAI, intraabdominal injection; IFN, interferon; Irf, interferon regulatory factor; Mφ, macrophage; MAPK, mitogen-activated protein kinase; MCP-1, monocyte chemotactic protein-1; MetS, metabolic syndrome; MIP1β, macrophage inflammatory protein-1β; NA, not applicable; Nos1, Nitric oxide synthase 1; Nfkb, nuclear factor kappa-light-chain-enhancer of activated B cells; NLRP3, nucleotide-binding oligomerization domain, leucine rich repeat and pyrin domain containing 3;OPN, osteopontin; PET-CT, positron emission tomography-computed tomography; PPARγ, peroxisome proliferator-activated receptor-gamma; Retnla, Resistin-like molecule alpha; ROS, reactive oxygen species; RP, Randall’s plaque; RTEC, renal tubular epithelial cell; SIRT3, sirtuin 3; TNFα, tumor necrosis factor alpha.;

### *In Vitro* and *Ex Vivo* Human Monocyte Function Against Calcium Oxalate Crystals in Kidney Stone Formers

Thongboonkerd et al. were the first to report the proteomic interactions between human monocytes and CaOx monohydrate (COM) crystals. The researchers found that COM could drive oxidative stress, resulting in increased cellular apoptosis and expression of prohibitin, plasminogen activator inhibitor-2, Alix, lamin A/C, and moesin, as well as reduced cellular survival, protein synthesis and stability, mRNA stability, and lipid metabolism. In addition, levels of La protein, heterogeneous nuclear ribonucleoprotein H1, elongation factor-2, otubain-1, heat shock protein (HSP) 105, and acyl-CoA thioester hydrolase were reduced (25). The proteomics associated with the monocyte functions evoked by COM in renal tubular epithelial cells (RTECs) have also been investigated. The researchers identified six secreted proteins with significantly altered expression levels in Mardin-Darby canine kidney (MDCK) cells incubated with COM crystals. Furthermore, they reported that enolase-1, a COM crystal-binding protein, activated monocytic cell migration by binding to the surface of monocytic cells (26).

Williams et al. further investigated monocyte mitochondrial function in human serum specimens and reported significantly reduced monocyte mitochondrial maximal respiration, reserve capacities, and bioenergetic health indices in patients with CaOx stones, as compared to healthy controls (27).

### *In Vitro* M (–) (Non-Polarized Macrophage) Phagocytosis and Calcium Oxalate Crystal Reaction

**Figure 2:**
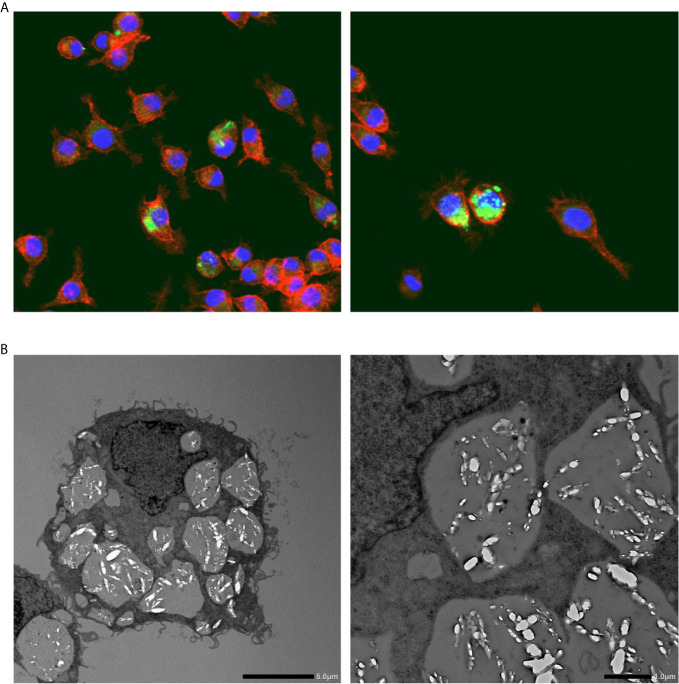
Calcium oxalate crystal phagocytosis by macrophages. **(A)** Representative photographs show fluorescent immunohistochemical staining of macrophages (RAW264.8) cultured with calcium oxalate monohydrate crystals. Red, phalloidin; Green: calcium oxalate monohydrate crystals; Blue, nucleus. Magnification: ×400. **(B)** Transmission electron microscopy images of macrophages (RAW264.8) engulfing calcium oxalate monohydrate crystals. Scale bars, left and right panels, 5 and 10 μm, respectively.

Confocal laser scanning microscopy revealed that I9.1 Mφs (derived from mouse spleen cells) that were co-incubated with macrophage colony-stimulating factor (M-CSF) dissolved internalized CaOx crystals (28). Okada et al. further investigated crystal dissolution by Mφs by using Lysotracker, transmission electron microscopy (TEM), and confocal light microscopy to study the murine J774.1 Mφ cell line. The authors confirmed the diachronic elimination of engulfed COM crystals in Mφs (**Figure 2**). Internalized COM crystals become surrounded by phagosomes that fuse with lysosomes to promote dissolution and spontaneous elimination (29). Mφ functions have also been investigated in RTEC. M-1 (murine collecting duct) and RAW264.7 cells incubated with murine Mφs developed into a pro-inflammatory state with increased COM crystal adhesion, especially when these cells were co-cultured with adipocytes. This paracrine mechanism is associated with increased mRNA and protein levels of OPN, MCP-1, and tumor necrosis factor (TNF)-α (30).

A previous study on Mφs derived from human monocytes incubated with COM crystals *in vitro* identified an association between HSP90 and F-actin on the phagosome membranes engulfing COM crystals. Blocking HSP90 with an siRNA reduced phagocytic activity and Mφ migration, suggesting that HSP90 and F-actin are involved in Mφ function during the CaOx interaction (31). Exosomes secreted by Mφs are also affected by COM crystals. In particular, COM-treated Mφ exosomes have increased membrane fragility and are able to enter the renal interstitium and trigger RTEC to release IL-8, which worsens tissue inflammation (32). Thus, COM-treated Mφ exosomes enhance monocyte and T-cell migration, monocyte activation, and Mφ phagocytosis. However, the suppression of vimentin with an siRNA abolishes these effects, suggesting that Mφ exosomal vimentin plays an important role in the immune response to COM crystals (33).

### *In Vivo* Macrophage Involvement in Calcium Oxalate Nephrocalcinosis

While there are limitations in adapting the findings to human patients owing to differences between human kidney stone formation and nephrocalcinosis in rodents, CaOx nephrocalcinosis models in rats and mice by hyperoxaluria are widely utilized for research, on the basis of similarities in intratubular crystal formation and retention (34). Mφs and multinucleated giant cells encapsulating interstitial CaOx crystals in both kidneys in a hyperoxaluric rat model and in patients with oxalosis were described in 1999 (17). The authors further confirmed that ectodermal dysplasia 1 (ED1)-positive Mφs were predominantly increased around renal crystal deposition sites compared with CD45 and major histocompatibility complex (MHC) class II positive and other mononuclear cells during the time course of hyperoxaluria in rats (35). These early studies showed that Mφ dissolve CaOx crystals, which might be related to renal defense against stone development. Okada et al. found spontaneously eliminated renal CaOx crystal deposition in a mouse model of nephrocalcinosis induced by glyoxylate (36), and that subsequent transcriptome studies associated the activation of monocytes/macrophages with this phenomenon involved chemokine (C-C motif) ligand 6, vimentin, Cd14, cytochrome P450 family 1 subfamily b polypeptide 1, moesin, apolipoprotein E, histocompatibility 2 class II antigen A α, histocompatibility 2 class II antigen A β 1, and lipopolysaccharide (LPS)-induced tumor necrosis factor. Immunohistochemical findings also confirmed that F4/80-positive Mφs could be visualized as crystal deposition peaks (37). The expression of monocyte chemoattractant protein (MCP)-1, osteopontin (OPN), fibronectin, cluster of differentiation (CD)44, and major histocompatibility complex (MHC) class II is associated with amounts of renal crystal deposition and the expression of F4/80-positive Mφs. TEM also revealed the phagocytosis of crystals by renal Mφ (38).

### *In Vitro* and *Ex Vivo* Polarized Macrophage Function Toward Calcium Oxalate Crystals

Examination of the roles of M1 and M2Mφs in CaOx crystals using murine bone marrow-derived Mφs revealed that M(IL-4) had a greater ability to phagocytose COM crystals than that does M(LPS) (39). In addition, M(IL-4+IL-13) suppresses COM crystal adherence to M-1 murine collecting duct cells and has higher COM-phagocytized cell rates than that does M(LPS+IFNγ) (19).

Yu et al. examined the role of Mφ in RP formation *in vitro* using human RTEC, HK-2 cells, with M(LPS) differentiated from the human monocytic cell line U937. They found that co-cultured M(LPS) and HK-2 cells incubated with hydroxyapatite increase oxidative stress, MCP-1 and OPN expression, and decrease fetuin-A (40). The same group assessed the role of M2Mφ in oxidative stress injury and apoptosis induced by CaOx crystals in HK-2 cells and found that M(IL-4+IL-13) differentiated from the human monocytic THP-1 cell line. Similar to apocynin, M(IL-4+IL-13) reduce the expression of NADPH oxidase p47phox protein, increase mitochondrial membrane potential, and inhibit the protein expression of cleaved caspase-3, cytochrome c, and phosphor-p38 MAPK, as well as ROS release in HK-2 cells incubated with CaOx crystals (41). These results indicated opposing roles of M1 and M2Mφs in the oxidative stress damage and apoptosis of RTEC during the development CaOx stones.

M(CSF-1) derived from human peripheral blood mononuclear cells demonstrate greater ability to phagocytose both CaOx crystals and natural kidney stones than that does M(GM-CSF). Inhibitor assays have demonstrated that kidney stone clearance is mediated through clathrin-dependent phagocytosis and endocytosis (42). Dominguez-Gutierrez et al. reported that CaOx, but not potassium or ZnOx, induced the M1-like Mφ differentiation of human monocyte cell lines and primary human monocytes expressing CD86 and CD68, and secrete cytokines and chemokines. Furthermore, supernatants of CaOx-treated monocytes can enhance M2Mφ CaOx crystal phagocytosis (43).

### *In Vivo* Polarized Macrophage Function in Renal CaOx Crystal Development

Several studies have assessed Mφ polarization in renal stone formation. In accordance with the mouse model of nephrocalcinosis with hyperoxaluria, more renal and urinary crystal deposition develops in CSF-1-deficient mice with fewer M2-like Mφs (identified as CD11b^+^F4/80^+^CD163^+^CD206^hi^ cells), than wild-type mice (39). In contrast, a surge in M1-like Mφs (identified as CD11b^+^F4/80^+^CD11c^int^Ly6C^hi^ cells) has been identified in MetS model mice with a leptin deficiency and CaOx crystal deposition in the kidney under treatment with ethylene glycol (EG), and fed with a high-fat diet (44). Further studies of the roles of M1 and M2Mφ roles in stone development have revealed that the induction/transfusion of M1- and M2-like Mφs respectively accelerated and attenuated renal crystal development in C57BL/6J wild-type mice administered daily with intra-abdominal glyoxylate (19). Anders et al. examined the role of the nucleotide-binding oligomerization domain, leucine-rich repeat, and pyrin domain-containing 3 (NLRP3), a central molecular mediator of inflammation in crystallopathies, in CaOx nephrocalcinosis formation in Nlrp3-deficient mice on a high-oxalate diet. They found that NLRP3 inhibition induces a shift in infiltrating renal Mφs from the M1-like (CD45^+^F4/80^+^CD11b^+^CX3CR1^+^ CD206^-^) to the M2-like (CD45^+^F4/80^+^CD11b^+^CX3CR1^+^CD206^+^TGFβ ^-^) phenotype and attenuation of renal fibrosis. Therefore NLRP3 appears to promote nephrocalcinosis-related fibrotic kidney disease by promoting a shift from anti-inflammatory M1, to pro-inflammatory and profibrotic M2-like Mφs (45).

### Roles and Polarization of Macrophage in Human Tissue

Microarray analysis of human kidneys revealed decreased expression of the M2-like Mφ-related genes, peroxisome proliferator- activated receptor gamma (PPARγ), CD163, and CD206, and increased expression of M1-like Mφ-related genes, including nitric oxide synthase 2, CSF2, IL10, and C-C chemokine receptor type 2, in renal papillary tissues from patients with CaOx stones compared with those who do not form stones (19). The findings of causal network analyses associated the differentially expressed genes in RP papillary tissues with significantly higher immune cell activity, including Mφs and plasma cells, which are linked to IL11, PG-endoperoxide synthase 1, glutathione peroxidase 3, and monocyte-to-Mφ differentiation in RP, than in normal papillary tissues (46). Urinary Mφ-related cytokines in stone-forming adolescents have also been investigated. Levels of urinary IL-13/creatinine and Mφ inflammatory protein-1β (MIP-1β)/creatinine could serve as useful biomarkers for stones based on sensitivity with 50% and 58%, respectively, and 93% specificity for both in a study of a small sample (47). Urinary multiplex comparisons among individuals who did not form stones, and those who formed CaOx stones for the first time and those who formed recurrent CaOx stones, identified IL-1a, IL-1b, IL-4, IL-10, and GM-CSF as potential biomarkers affecting Mφ and neutrophil function in stone development (48). Furthermore, M1-like Mφ polarization increases pro-inflammatory cytokines such as TNFα, IL-1β, and IL-1, as well as M1/M2-like monocyte ratios in blood samples from patients with CaOx stones compared with those who do not form stones (49).

### Therapeutic Target Altering Macrophage Phenotype Against Kidney Stone Disease

Sirtuin 3 (SIRT3), an NAD^+^-dependent deacetylase in the mitochondrial matrix, suppresses renal CaOx crystal deposition *in vitro* and *in vivo* by promoting M2-like Mφs through deacetylating forkhead box O1 (FOXO1) (49). The prevalence of kidney stones is influenced by sex hormones (47). Suppressing androgen receptor (AR) expression in RTEC *in vitro* increases Mφ recruitment and causes an M2 polarization shift, which increases the phagocytosis of intrarenal CaOx crystals (47). Administering renal tubule-specific AR knockout mice and hydroxy-L-proline treated rats with an AR degradation enhancer *in vivo* has revealed that AR signaling suppresses CSF-1 expression *via* the upregulation of miR-185-5p, which results in decreased M2-like Mφs and accelerated CaOx crystal development (50). The potential of pioglitazone, a PPARγ agonist, to treat kidney stones has been recognized (51, 52). Pioglitazone increases M2-like Mφ polarization and decreases renal CaOx crystal deposition and inflammatory damage and in murine bone marrow-derived Mφ *in vitro* and in CaOx nephrocalcinosis mouse models *in vivo*. These results indicated that PPARγ upregulates miR-23 expression and subsequently attenuates the expression of interferon regulatory factor 1 (Irf1) and Pknox1, which shifts the Mφ phenotype from M1 to M2 (53). The effects of nuclear factor erythroid 2-related factor 2 (Nrf2) on PPARγ and the anti-inflammatory process in individuals with CaOx stone have been investigated. The findings showed that Nrf2 attenuates the M1-like Mφ polarization shift by suppressing toll-like receptor 4 (TLR4) and IRF *in vitro*. Moreover, sulforaphane, an activator of Nrf2, plays a protective role against CaOx crystal formation and renal injury *via* the Nrf2-miR-93-TLR4/IRF1 axis, which promotes M2-like Mφ polarization and inhibits RTEC inflammation *in vivo* in mouse models of CaOx nephrocalcinosis (54). The involvement of the aryl hydrocarbon receptor (AhR) as a regulator of the phenotypic balance between M1- and M2-like Mφs in renal CaOx stone development has been investigated (55). Transcriptomic and proteomic analyses of bone marrow-derived Mφs and mouse models of CaOx nephrocalcinosis have revealed that stimulating the AhR-miR-142a-IRF1/hypoxia-inducible factor (HIF)-1α axis diminishes M1-like Mφs and promotes M2-like Mφs, leading to the suppression of renal CaOx crystal deposition and stone-related renal damage (55).

## Limitations

This systematic review has several limitations. Firstly, most articles we found utilized the M1/M2Mφ definition to be consistent with the prior literature in the nephrology and urology fields; therefore, in some cases it was difficult to summarize them according to the latest nomenclature. Secondly, findings from nephrocalcinosis mouse models might not be applicable to kidney stone patients owing to pathological differences between intratubular crystal deposition and CaOx stone formation. Lastly, only a few studies have assessed the direct relationship between CaOx stones/crystals and Mφs in human tissues; thus, additional live tissue studies are required.

## Conclusion

**Figure 3:**
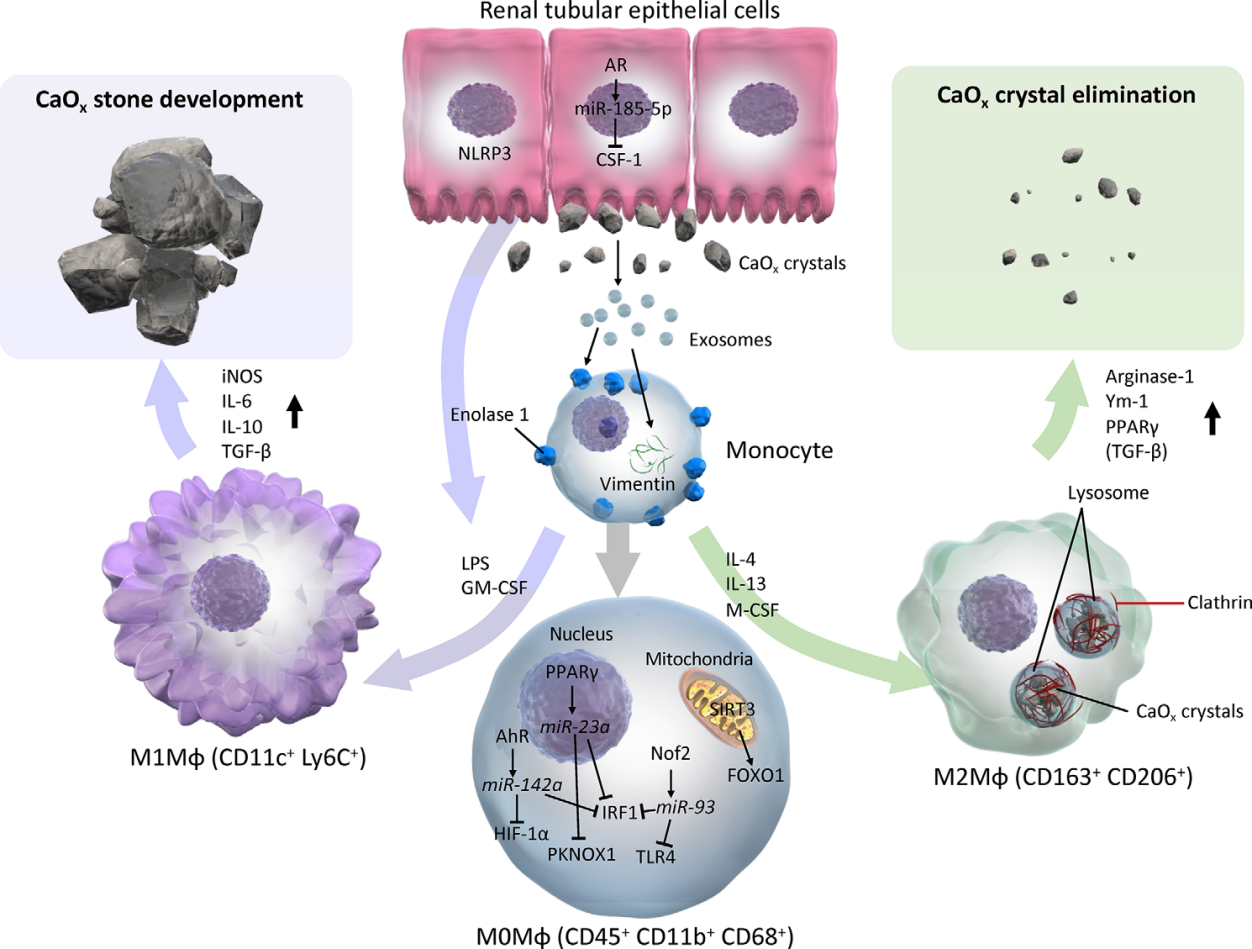
Schema of evidence synthesized from current literature regarding roles of M1 and M2 macrophages in CaOx stone development. M1 or M2Mφs are usually differentiated from monocytes and M0 (with neutral polarization) Mφs *via* various cytokines/chemokines by direct and indirect influences from CaOx crystals. M0 Mφs autocrine mechanism shifts phenotypes toward CaOx crystal development *via* AhR-miR-142a-IRF1/HIF-1α, PPARγ-miR-23a-IRF1/PKNOX1, Nrf2-miR-93-IRF/TLR4, and SIRT3-FOXO1 axes. Monocytes that reflect exosomes secreted by monocytes/Mφs and renal tubular epithelial cells *via* enolase-1 and vimentin, then become activated and change into either M1 or M2Mφs. In contrast, paracrine involvement of renal tubular epithelial cells *via* NLRP3 and AR-miR-185-5p-CSF-1 is also an important factor in regulation of Mφ polarization. While M1Mφs facilitate CaOx stone development by promoting pro-inflammatory and oxidative stress molecules such as iNOS, IL-6, IL-10, and TGF-β, M2Mφs attenuate the development of CaOx crystals, and eliminate them by phagocytosis *via* lysosomes and clathrin mediation, and induces anti-inflammatory molecules including Arginase-1, Ym-1, and PPARγ. AhR, aryl hydrocarbon receptor; AR, androgen receptor; CSF-1, colony-stimulating factor-1; FOXO1, forkhead box O1; GM-CSF, granulocyte-macrophage colony-stimulating factor; HIF-1α, hypoxia-inducible factor-1 alpha; iNOS, inducible nitric oxide synthase; IRF1, interferon regulatory factor 1; LPS, lipopolysaccharide; Mφ, macrophage; M-CSF, macrophage colony-stimulating factor; NLRP3, nucleotide-binding oligomerization domain, leucine-rich repeat, and pyrin domain containing 3; PPARγ, peroxisome proliferator-activated receptor-gamma; SIRT3, Sirtuin 3; TLR4, toll-like receptor 4.

The roles of Mφs in the development of CaOx kidney stone have been investigated *in vitro*, *in vivo*, and in human specimens *ex vivo. In vitro* and *ex vivo* studies have demonstrated that monocytes and M(-) (non-polarized Mφs) have the ability to eliminate CaOx crystals *via* phagocytosis. The treatment of RTECs with COM crystals stimulates Mφ migration *via* cytokines, chemokines, and exosomes. *In vivo* studies have shown M1-like Mφs facilitate renal CaOx crystal development with renal inflammation, fibrosis, and cellular damage, whereas M2-like Mφs suppress CaOx crystal development. M2-like Mφs, such as M(CSF-1), M(IL-4), and M(IL-4+IL-13), have greater capability for phagocytosing CaOx crystals, which eventually dissolves crystal fragments, and autocrine and paracrine mechanisms along with RTEC enhance Mφ phagocytosis (**Figure 3**). Furthermore, while only a few studies have investigated Mφ polarization in human tissues, there may be a predominant M1-like Mφ cytokine/chemokine phenotype in the urine, serum, and renal tissues of kidney stone formers.

Research focus has shifted from identifying Mφ expression to analyzing Mφ function and finding triggers that alter the Mφ phenotypes to create novel therapeutic targets. However, evidence regarding Mφs in urinary sediments is scant, and the direct manipulation of Mφ phenotypes for clinical use needs to be determined (56). Future investigations should strive to establish a urinary biomarker for liquid biopsies (57), and an Mφ-specific target therapy using antibodies, vectors, and nanoparticles (58, 59).

## Author Contributions

KT, SH, and TY: Study conception and design. KT and RU: Data collection. KT, RU, and AO Data analysis. KT, SH, and AO: Data interpretation. KT and RU: Drafted the manuscript. SH, AO, and TY: Revised the manuscript. TY supervised the study. All authors contributed to the article and approved the submitted version.

## Funding

This work was supported in part by Grants-in-Aid for Scientific Research from the Ministry of Education, Culture, Sports, Science and Technology, Japan (nos. 19H03791, 19K09735, and 20K21658), as well as grants from the NOVARTIS Foundation (Japan), the Naito Foundation, and the Hori Sciences and Arts Foundation.

## Conflict of Interest

The authors declare that the research was conducted in the absence of any commercial or financial relationships that could be construed as a potential conflict of interest.

## Abbreviations

AhR, aryl hydrocarbon receptor; AR, androgen receptor; CaOx, calcium oxalate; COM, calcium oxalate monohydrate; CSF-1, colony stimulating factor-1; EG, ethylene glycol; FOXO1, forkhead box O1; GM-CSF, granulate macrophage colony stimulating factor-1; HIF-1α, hypoxia-inducible factor-1 alpha; HLP, hydroxy-L-proline; IFNγ, interferon gamma; Irf1, interferon regulatory factor 1; LPS, lipopolysaccharide; MAPK, mitogen-activated protein kinase; MCP-1, monocyte chemotactic protein-1; MetS, metabolic syndrome; MHC, major histocompatibility complex; MIP1β, macrophage inflammatory protein-1β; Mφ, macrophage; NLRP3, nucleotide-binding oligomerization domain, leucine rich repeat and pyrin domain containing 3; Nrf2, nuclear factor erythroid 2-related factor 2; OPN, osteopontin; PPARγ, peroxisome proliferator-activated receptor-gamma; HSP, heat shock protein; PRISMA, Preferred Reporting Items for Systematic Reviews and Meta-Analyses; ROS, reactive oxygen species; RP, Randall plaque; RTEC, renal tubular epithelial cell; SIRT3, sirtuin 3; TLR4, toll-like receptor 4; TNFα, tumor necrosis factor alpha.
